# Nephrotoxicity-induced proteinuria increases biomarker diagnostic thresholds in acute kidney injury

**DOI:** 10.1186/s12882-017-0532-7

**Published:** 2017-04-03

**Authors:** Fahim Mohamed, Nicholas A. Buckley, John W. Pickering, Klintean Wunnapuk, Sandamali Dissanayake, Umesh Chathuranga, Indika Gawarammana, Shaluka Jayamanne, Zoltan H. Endre

**Affiliations:** 1 South Asian Clinical Toxicology Research Collaboration, University of Peradeniya, Peradeniya, Sri Lanka; 2 Department of Pharmacy, Faculty of Allied Health Sciences, University of Peradeniya, Peradeniya, Sri Lanka; 3 Department of Nephrology, Prince Of Wales Hospital and Clinical School, University of New South Wales, Sydney, Australia; 4 TACT Research Group, Department of Pharmacology, SOMS, Sydney Medical School, University of Sydney NSW, Sydney, Australia; 5 Department of Medicine, University of Otago Christchurch, Christchurch, New Zealand; 6 Emergency Department, Christchurch Hospital, Christchurch, New Zealand; 7 Department of Forensic Medicine, Faculty of Medicine, Chiang Mai University, Chiang Mai, Thailand; 8 SACTRC, Faculty of Medicine, University of Peradeniya, Peradeniya, Sri Lanka

**Keywords:** Paraquat, Poisoning, Albuminuria, Biomarkers, Nephrotoxicity

## Abstract

**Background:**

Paraquat ingestion is frequently fatal. While biomarkers of kidney damage increase during paraquat-induced acute kidney injury (AKI), significant concurrent proteinuria may alter diagnostic thresholds for diagnosis and prognosis to an unknown extent. This study evaluated the effect of albuminuria on biomarker cutoffs for diagnosis and outcome prediction.

**Methods:**

This was a multi-centre prospective clinical study of patients following acute paraquat self-poisoning in 5 Sri Lankan hospitals. Biomarker concentrations were quantified using ELISA and microbead assays and correlated with urinary albumin. Functional-AKI was defined by the Acute Kidney Injury Network serum creatinine definition and alternatively by a ≥50% increase in serum cystatin C. Albuminuria was defined as albumin-creatinine ratio >30 mg/g. The study outcomes were compared with a retrospective analysis of a pre-clinical study of paraquat-induced nephrotoxicity with appropriate controls.

**Results:**

Albuminuria was detected in 34 of 50 patients, and increased with functional-AKI severity. The concentrations of uNGAL, uCysC, uClusterin, uβ2M, and uKIM-1 were higher in albuminuric compared to non-albuminuric patients (*p* < 0.001). Albuminuria correlated with biomarker concentration (r > 0.6, *p* < 0.01) and was associated with death (*p* = 0.006). Optimal biomarker cutoffs for prediction of death were higher in the albuminuric group. Similar outcomes with more detailed analysis were obtained in experimental paraquat nephrotoxicity.

**Conclusion:**

Albuminuria was associated with paraquat-induced nephrotoxicity and increased excretion of low-molecular weight protein biomarkers. AKI biomarker cutoffs for diagnosis, outcome prediction and AKI stratification increased in the presence of albuminuria. This may lead to over-diagnosis of AKI in conditions independently associated with proteinuria.

**Electronic supplementary material:**

The online version of this article (doi:10.1186/s12882-017-0532-7) contains supplementary material, which is available to authorized users.

## Background

Acute kidney injury (AKI) is common and has diverse aetiology [1–3]. Nephrotoxic drugs are common contributory factors to AKI [4]. In Asia, purely nephrotoxic AKI (ToxAKI) is commonly seen following deliberate ingestion of agrochemicals [5–8].

AKI definitions have evolved around changes in creatinine or urine output [9, 10], with both measures lacking specificity and sensitivity for early AKI detection. Furthermore, plasma creatinine concentrations usually respond only slowly to kidney damage and may be altered by non-renal mechanisms [11, 12]. Alternative strategies for defining AKI with kidney-specific structural (injury) biomarkers have been proposed which may diagnose AKI earlier and with greater specificity and sensitivity than creatinine [13–17]. However, structural biomarker-based definitions also carry several challenges. One of these is low or absent biomarker concentrations in healthy populations. If these are normally absent, then the appearance of any biomarker should herald disease. Alternatively, reference ranges need to be defined for healthy populations and in the presence of co-morbidities. The majority of studies report biomarker reference ranges in heterogeneous ill subjects without AKI [18–20] and only a few studies define (some) biomarker concentrations in healthy populations [21, 22]. In addition, non-renal factors that increase structural biomarker concentrations independently of renal injury, have not yet been incorporated into AKI definitions [21–25]. In particular, proteinuria and albuminuria, both important biomarkers, increase excretion of urinary Neutrophil gelatinase-associated lipocalin (NGAL), and urinary cystatin C (uCysC) in critically ill patients [26].

Our observation of significant proteinuria following paraquat poisoning prompted analysis of the influence of proteinuria on the excretion of other renal biomarkers. We hypothesised that paraquat-induced albuminuria would increase the excretion of low molecular weight protein biomarkers subject to tubular reabsorption. This was examined in a prospective clinical study of patients following acute paraquat ingestion and then in a retrospective analysis of controlled data in an experimental rodent model of paraquat nephrotoxicity. These studies determined the effect of albuminuria on biomarker cutoffs for AKI diagnosis and outcome prediction.

## Methods

### Clinical study

This multi-centre prospective observational study was approved by the human research ethics committees of both the University of New South Wales (Sydney) and University of Peradeniya (Sri Lanka). The recruitment of healthy controls was carried out in 3 major provinces (North Central, Central and Southern Province of Sri Lanka) where the ‘Sinhala’ ethnic group is predominant; our patient cohort was also recruited from the hospitals located in these regions. These areas were selected since a high incidence of self-poisoning is reported from these regions. Healthy volunteers from several regions of Sri Lanka (outside the chronic kidney disease of unknown origin areas) were asked to volunteer for this study and informed written consent was obtained. All consenting volunteers underwent clinical screening and patients who had a history of any existing clinical conditions were excluded from this cohort. Single blood and urine samples were collected to quantify normal biomarker concentrations. Detail methods of patient recruitment to this cohort is described elsewhere [12, 27] and the patient cohort studied in this manuscript is identical to that discussed previously [12, 27]. Briefly, patients who presented to adult medical units of 5 study hospitals with a history of paraquat self-poisoning within 24 h of ingestion were consented for inclusion in the study. Patients who were young (age <15 years), or co-ingested another chemical were excluded. Informed written consent was obtained from all patients or their accompanying relatives. Paraquat ingestion was confirmed by a positive urine dithionate test four hours after ingestion. Demographic, clinical, and laboratory data were collected prospectively until discharge. Blood and urine sampling was scheduled at 4, 8, 16, 24 h after ingestion and then daily. Collected samples were immediately processed and stored in −70 °C freezers until batch-wise assays commenced.

### Pre-clinical study

The rodent study was approved by the University Animal Ethics Committee (Health Sciences) of the University of Queensland and conducted as part of a doctoral study by one of the authors, KW at Therapeutic Research Centre, University of Queensland, Australia. Raw biomarker data [urinary albumin (uAlb), kidney injury molecule-1 (uKIM-1), cystatin C (uCysC), clusterin (uClu), osteopontin (uOstP), neutrophil gelatinase-associated lipocalin (uNGAL), beta-2-microglobulin (uβ2M)] from that study [28] was used to evaluate the influence of urinary albumin on the excretion of urinary biomarkers. Detailed methods including animal handling, paraquat dose, sample collection, biomarker assays, and histopathology of the original study are described elsewhere [28]. Briefly, male Wistar rats (200–250 g) from the Animal Resources Centre (Western Australia, Australia) were housed on a 12 h light/dark cycle. The animals were allowed free access to food (standard laboratory chow) and water. Rats were fasted overnight (12 h) before the experiments. Control rats (*n* = 6) were gavaged with water. The treatment group rats were randomly divided into the 4 dose groups with 6 rats in each group. These were dosed orally with 4 different doses (15, 30, 60 and 90 mg/kg) of paraquat dichloride solutions (Sigma-Aldrich, St. Louis, MO, USA). These doses were approximately 10, 25, 50, and 70% of the LD50 in rats [28]. After administration, rats were housed in individual metabolic cages and urine samples collected on dry ice at intervals of 0–8 h, 8–24 h and 24–48 h. Blood was collected from the tail vein at 8 and 24 h. At 48 h, the rats were sacrificed and blood was collected from the vena cava. Histopathology grades of 1 to 7 (1-no changes, 7-severe) were assigned for each rat [28] and represent the total numbers of necrotic cells and pyknotic nuclei in paraquat-treated rats normalised to total baseline necrotic cells and pyknotic nuclei counted in the control groups and assigned by KW under the supervision of two pathologists.

#### Biomarker assays

Serum creatinine (sCr) was measured using the Jaffe method (kinetic Jaffe reaction method, rate blank and compensated) on Roche Hitachi 912 automatic analyser. DuoSet ELISA development kits supplied by R&D systems^®^ were used for quantifying uKIM-1, and uClu. Urinary interleukin-18 (IL-18) was measured using a commercially available ELISA kit (Bender MedSystems GmbH, Vienna, Austria). Intra and inter assay precision for ELISA was < 10%. Other AKI biomarkers such as uCysC, uAlb, trefoil factor-3 (uTFF3), uOstP, uβ2M and uNGAL were quantified on the same sample using Bio-Plex Pro™ RBM Human Kidney Toxicity Assays panel 2 on the Bio-Plex 200 system (BIO-RAD). Inter and intra assay precision estimates were <15% and <5% respectively. Rodent samples were assayed similarly as previously described [28].

#### Outcome definition and statistical analysis

Biomarker concentrations were reported as absolute concentrations and as normalised to urinary creatinine concentration. Albuminuria in patients was defined as urinary albumin creatinine ratio [(ACR) ≥30 mg/g (μg/mg)] [29, 30]. Albuminuria in the pre-clinical study was defined as ACR values ≥ 95^th^ centile from control rats since cut-offs for albuminuria in rats were not available. Functional-AKI was defined by change in sCr according to the Acute Kidney Injury Network (AKIN) [10] criteria or ≥50% change in serum cystatin C [31]. Moderate to severe functional-AKI was defined as an increase in sCr of ≥200% (AKIN Stage 2) or 300% (AKIN Stage 3) respectively [32]. The 95^th^ centile of each structural biomarker obtained in healthy volunteers was defined as the cutoff for structural-AKI. This definition was modelled on similar approaches to define healthy reference cutoff points of cardiac troponin for diagnosis and risk stratification of myocardial injury [33, 34].

Continuous variables were compared using the Wilcoxon rank sum test and reported as median and interquartile range. Categorical variables were reported as proportions and compared using Fisher’s exact test. Correlations were done using the Spearman rank-order. The prognostic performance of each biomarker for predicting death was evaluated by area under the receiver operating characteristic curves (AUC-ROC) stratifying to albuminuria and the optimal threshold for each biomarker was calculated. For each biomarker, the sensitivity and specificity of structural-AKI for predicting functional-AKI or death was calculated separately in the presence or absence of albuminuria. The statistical analyses were conducted using GraphPad Prism version 6 (GraphPad Software, San Diego, USA) and STATA IC10 (StataCorp, 2007).

## Results

### Clinical study findings

#### Biomarker concentrations in healthy volunteers

Plasma and urinary functional and urinary structural injury biomarker levels in urine and blood samples from 63 healthy young adult volunteers [median age 28 years (IQR 26–33), 70% male] are presented as absolute (Table 1) and normalised concentrations (Additional file 1: Table S1).

**Table 1:** Serum and urinary functional and injury biomarkers in healthy subjects (absolute concentrations)

Biomarkers	Median and IQR	Lower reference limit (5^th^ centile)	Upper reference limit (95^th^ centile)
*Serum biomarkers*			
Total protein (g/dl)	6.5 (5.8–7.1)	5.3	8.9
Albumin (g/dl)	4.1 (3.8–4.3)	3.2	5.3
Creatinine			
mg/dl	0.82 (0.72–0.94)	0.56	1
μmol/L	72 (64–84)	50	88
Cystatin C (mg/l)	0.84 (0.78–0.88)	0.6	1
*Urinary biomarkers*			
Total protein (mg/L)	196 (161–267)	70	1870
Creatinine			
mg/dl	123 (54–182)	33	253
mmol/L	11 (5–16)	3	22
Urea			
mg/dl	1432 (822–2023)	444	3206
mmol/l	511 (294–722)	159	1144
Cystatin C (ng/ml)	24 (11–51)	3.8	94
Albumin (ng/ml)	5700 (1700–9200)	500	21000
NGAL (ng/ml)	14.8 (10.2–40.3)	3.4	134
KIM-1 (ng/ml)	0.57 (0.33–1.23)	0.02	2.8
Clusterin (ng/ml)	217 (104–393)	28	798
β2M (ng/ml)	67 (36–139)	14.5	250
Osteopontin (ng/ml)	1400 (400–0.2800)	100	6800
TFF3 (ng/ml)	1400 500–1500)	200	3200
IL-18 (pg/ml)	53.8 (39–80)	39	136

#### Patient baseline demographics according to ACR

The 50 confirmed paraquat-poisoned patients were previously healthy young adults [median age, 24 years (IQR 19–32, range 15–56)], and 55% male. Baseline demographic and clinical characteristics were similar for albuminuric and non-albuminuric groups (Table 2) except for serum creatinine and paraquat concentrations. Thirty-four (70%) developed albuminuria within 16–24 h. Twenty-six patients developed moderate to severe functional-AKI (AKI stage 2, *n* = 7, or stage 3, *n* = 19). The incidence of moderate to severe functional-AKI was higher in albuminuric patients (68% compared with 25%; *p* < 0.01). Based on the serum cystatin C definition of functional-AKI, 19 patients developed functional-AKI, of these 17 had albuminuria. All 12 deaths occurred in patients with albuminuria (*p* < 0.01).

**Table 2:** Baseline demographic and clinical characteristics

Baseline characteristics	No-albuminuria (ACR < 30 mg/g) (*n* = 16)	Albuminuria (ACR ≥ 30 mg/g) (*n* = 34)	*p*
Age (years)	23 (19–35)	25 (19–32)	0.97
Male gender (%)	60	50	0.5
Weight (kg)	50 (45–55)	50 (39–60)	0.65
Volume ingested (ml)	10 (5–30)	20 (20–50)	0.05
Time to admission (hours)	4 (2–6)	3.5 (2–7.5)	0.98
Pulse (beats/min)	80 (78–88)	82 (80–89)	0.50
BP systolic (mm Hg)	120 (110–120)	110 (110–120)	0.18
BP diastolic	80 (70–80)	70 (70–80)	0.52
sCr (mg/dl)	0.7 (0.5–0.8)	0.9 (0.7–1.3)	0.006
sCysC (mg/l)	0.7 (0.6–0.8)	0.7 (0.6–0.8)	0.53
Maximum serum paraquat (ng/ml/24 h)	20 (10–120)	640 (140–1400)	0.0006
Functional-AKI (%)	8 (50%)	29 (85%)	0.007
Death (n)	0	12	0.006

#### Albuminuria and functional AKI

**Fig. 1:**
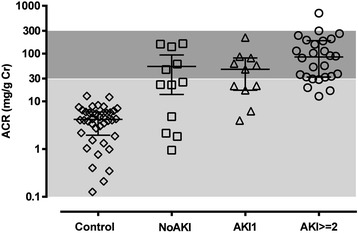
ACR in healthy controls and patients with or without functional-AKI. Albuminuria (*dark grey shaded area*; ACR ≥ 30 mg/g); normal ACR (*light grey shaded area*, <30 mg/g). AKI is defined based on AKIN classification

ACR increased with functional AKI severity (*p* < 0.0001, Fig. 1) and was greater in patients with moderate to severe functional-AKI compared to the healthy controls (*p* < 0.0001). Albuminuria was also observed in 5 patients who did not develop functional-AKI (based on either sCr or sCysC definition) but did have increased structural biomarker concentrations. The median ACR was higher in paraquat-poisoned subjects without AKI compared to the healthy controls (*p* < 0.05) (Fig. 1).

#### Biomarker concentrations stratified by ACR and AKI

Within 24 h of paraquat ingestion, maximum (normalised) biomarker concentrations were increased in the presence of albuminuria. In albuminuric patients, uCysC, uClu, uNGAL, uKIM-1, and uβ2M increased with increasing AKI severity (Additional file 2: Figure S1). Concentrations were lower in patients who did not develop albuminuria. The concentrations of filtered biomarkers, uCysC, uNGAL, uClu, uβ2M, uTFF3 and uKIM-1 were higher in albuminuric patients than healthy controls (*p* < 0.001) (Additional file 2: Figure S1).

#### Correlation of urinary biomarkers and albuminuria

**Fig. 2:**
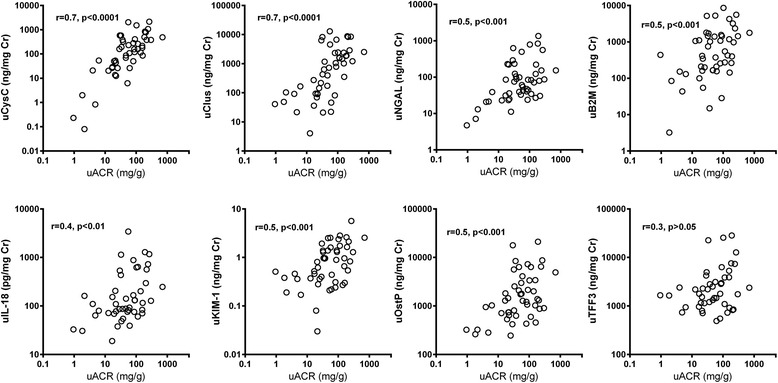
Correlation between normalised biomarker concentration and ACR following paraquat poisoning

Normalised uCysC and uClu correlated well with ACR (r = 0.7, *p* < 0.0001). Urinary NGAL, β2M, OstP and KIM-1 also correlated with ACR (r = 0.5, *p* < 0.01). Correlations with ACR for uIL-18 (r = 0.4, *p* < 0.05) and uTFF3 (r = 0.3, *p* > 0.05) were modest (Fig. 2). A similar correlation profile was obtained when the absolute urinary concentration of each biomarker was assessed against absolute uAlb (Additional file 2: Figure S2).

#### Biomarker threshold for predicting death as an outcome in the presence of albuminuria

Twelve (*n* = 12) patients died during the hospital stay and all had developed albuminuria. No patient without albuminuria died (*p* < 0.01). Table 3 summarises the AUC-ROCs, specificity, sensitivity and the optimal cutoff for each biomarker to predict death in the albuminuric group (*n* = 34, 12 deaths) and for the entire patient cohort (*n* = 50, 12 deaths). There was an almost 2-fold increase in biomarker cut-offs for uCysC, uClu and uβ2M in the albuminuric cohort with smaller increases in the cutoffs for the other biomarkers (Table 3). In addition, biomarker performance was uniformly reduced in the presence of albuminuria.

**Table 3:** Comparative diagnostic performance of renal biomarkers in predicting death in paraquat poisoning stratified by albuminuria

All patients (*n* = 50)					Patients with albuminuria (*n* = 34) ^a^				*p* ^‡^
Biomarkers (ng/mg Cr)	AUC-ROC (95% CI)	Cut-off	Sensitivity (95% CI)	Specificity (95% CI)	AUC-ROC (95% CI)	Cutoff	Sensitivity (95% CI)	Specificity (95% CI)	
uCysC	0.78 (0.62–0.94)	>200	67 (35–90)	67 (50–82)	0.68 (0.49–0.88)	>300	67 (35–90)	66 (43–84)	0.46
uClu	0.70 (0.52–0.87)	>750	67 (34–90)	60 (42–75)	0.55 (0.34–0.76)	>1600	58 (28–74)	59 (36–80)	0.29
Uβ2M	0.68 (0.48–0.88)	>990	67 (35–90)	68 (50–82)	0.60 (0.39–0.82)	>1280	67 (35–90)	68 (45–86)	0.62
uNGAL	0.81 (0.67–0.95)	>80	67 (35–90)	68 (50–82)	0.75 (0.56–0.93)	>90	67 (35–90)	68 (45–86)	0.60
uKIM-1	0.75 (0.57–0.90)	>0.96	75 (43–94)	73 (56–85)	0.61 (0.41–0.82)	>1.3	67 (35–90)	40–83)	0.38
uTFF3	0.85 (0.72–0.98)	>2340	75 (43–94)	71 (52–85)	0.82 (0.67–0.97)	>2830	75 (43–94)	70 (46–88)	0.74
uOstP	0.82 (0.68–0.97)	>1760	75 (42–94)	70 (53–84)	75 (57–94)	>2100	75 (42–94)	68 (45–86)	0.60
uIL-18 (pg/mg Cr)	64 (45–82)	>130	63 (31–89)	60 (42–75)	55 (34–76)	>130	63 (31–89)	50 (28–72)	0.54

^a^ Albuminuria; ACR ≥ 30 mg/g, All normalised biomarker concentrations are presented in ng/mg Cr except uIL-18 (pg/mg Cr)

^‡^The AUC-ROC values were compared using Delong method

#### Sensitivity and specificity of structural biomarker based definition in diagnosis of AKI

**Fig. 3:**
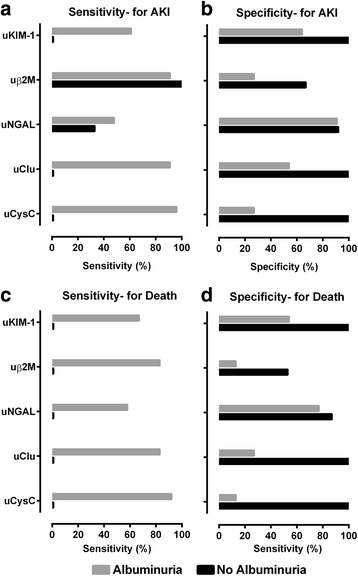
Sensitivity and specificity of 95^th^ centile values of structural biomarker values from healthy volunteers in detecting functional-AKI or death. Serum creatinine ≥ 100% (AKI ≥ 2) is defined as functional-AKI while biomarker concentration >95th centile value in healthy volunteers (uCysC: 70 ng/mg Cr; uClu: 420 ng/mg Cr; uKIM-1 1.2 ng/mg Cr; uβ2M 166 ng/mg Cr and uNGAL: 120 ng/mg Cr) were used to define structural-AKI. *Grey* and *black* area on the chart depicts 'Albuminuria' and 'No albuminuria' respectively

The sensitivity of the 95^th^ centile in the healthy volunteer group of structural biomarkers for diagnosis of moderate to severe functional-AKI was high amongst albuminuric patients for both uCysC (sensitivity 96%, CI 79-99%; diagnostic odds ratio = 8, CI 1–91) and uClu (sensitivity 91%, CI 73–98%; diagnostic odds ratio = 13, CI 2–82) (Table 4). Similarly, the sensitivity of structural AKI for predicting death was higher for both uCysC (sensitivity 92%, CI 64–98%; diagnostic odds ratio = 2, CI 0.2–18) and uClu (sensitivity 83%, CI 55–95%; diagnostic odds ratio = 2, CI 0.3–11) in patients with albuminuria (Fig. 3, Table 4). But, the lower end of the confidence interval for the odds ratios for AKI diagnosis using both biomarkers in predicting mortality was less than 1. Furthermore, the specificity of these two biomarkers in diagnosing functional AKI or predicting death was low (<30%) in patients with albuminuria. In contrast, the sensitivity for predicting death or diagnosing functional AKI by uCysC or uClu was low (1%) in non-albuminuric patients (Fig. 3, Table 4).

**Table 4:** Sensitivity and specificity of 95^th^ centile values of structural biomarker values from healthy volunteers in detecting functional-AKI

	Patients with albuminuria (*n* = 34)					No-albuminuria (*n* = 16)				
Biomarkers (ng/mg Cr)	Sensitivity^a^	Specificity^a^	Positive likelihood ratio^a^	Negative likelihood ratio^a^	Diagnostic odds ratio^a^	Sensitivity^a^	Specificity^a^	Positive likelihood ratio^a^	Negative likelihood ratio^a^	Diagnostic odds ratio^a^
uCysC	96 (79–99)	27 (10–56)	1.3 (0.9–1.9)	0.16 (0–1.4)	8.2 (0.7–91.2)	0 (0–56)	100 (75–100)	0	1 (1–1)	0
uClu	91 (73–98)	54 (28–79)	2 (1–3.4)	0.2 (0–0.7)	12.6 (1.9–82)	0 (0–56)	100 (75–100)	0	1 (1–1)	0
Uβ2M	91 (73–98)	27 (9–56)	1.2 (0.8–1.8)	0.3 (0–1.6)	3.9 (0.5–28)	100 (43–100)	66 (39–86)	3 (1.3–6.7)	0	0
uNGAL	48 (29–57)	91 (62–98)	5.3 (0.8–35.8)	0.6 (0.4–0.9)	9.2 (1–83)	33 (6–79)	92 (64 (98)	4 (0.3–47)	0.7 (0.3–16)	5.5 (0.2–129)
uKIM-1	60 (40–78)	63 (35–85)	1.7 (0.7–3.9)	0.6 (0.3–1.2)	2.7 (0.6–12)	0 (0–56)	1 (75–100)	0	1 (1–1)	0

^a^ Data presented with 95% CI

Serum creatinine ≥ 100% (AKI ≥ 2) is defined as functional-AKI while biomarker concentration >95th centile value in healthy volunteers (uCysC: 70 ng/mg Cr; uClu: 420 ng/mg Cr; uKIM-1 1.2 ng/mg Cr; uβ2M 166 ng/mg Cr and uNGAL: 120 ng/mg Cr) were used to define structural-AKI

Urinary β2-M structural AKI displayed excellent sensitivity for diagnosing functional-AKI in both albuminuric (sensitivity 91%, CI 73–98%; diagnostic odds ratio = 4, CI 0.5–21) and non-albuminuric subjects (sensitivity 100%, CI 44–100%). Among 5 albuminuric patients who did not develop functional-AKI (Fig. 3, Table 4), all had structural AKI based on sufficient increases in at least one damage biomarker.

### Preclinical study findings

#### Albuminuria and biomarkers in paraquat treated rats

**Fig. 4:**
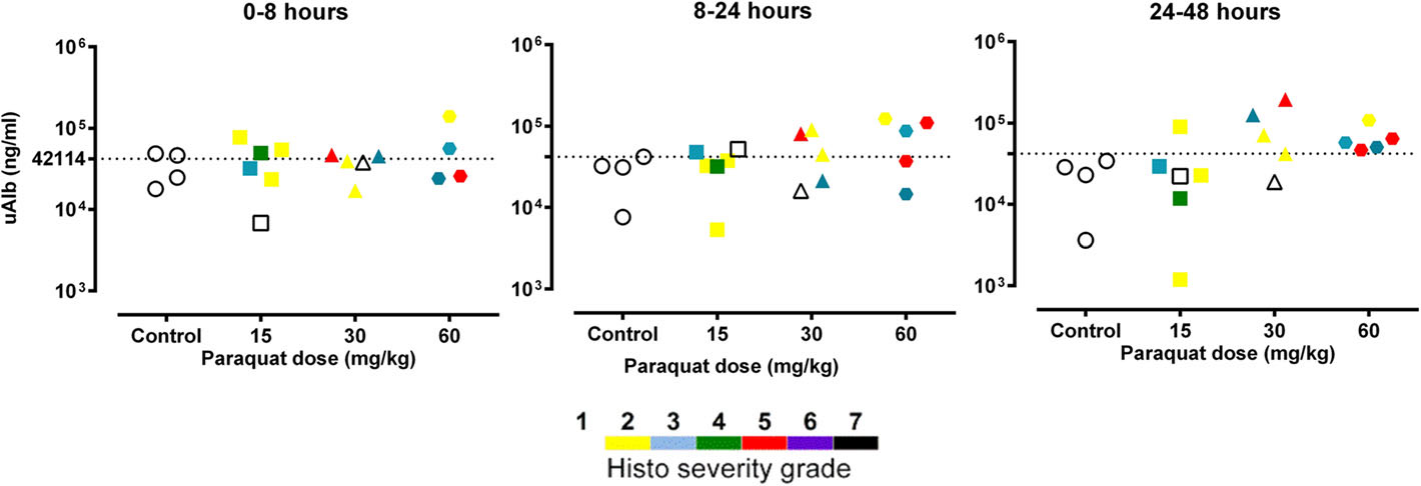
Urinary albumin concentrations at different time points in controls and paraquat treated rats. Note that albumin concentration (*y axis*) values are actual raw values (*not logarithmic values*). Y axis is formatted on log scale to improve the visibility of data points. An increase urinary albumin concentration was observed as histopathology grades increased in paraquat treated rats. Each symbols indicate biomarker concentrations from individual rats at specific paraquat dose levels. The histopathology grades [28] are displayed on a scale of 0 (*normal*) to 7 (*severe*) [grade 1 (*white*), grade 2 (*yellow*), grade 3 (*blue*), grade 4 (*green*), grade 5 (*red*), grade 6 (*purple*) and grade 7 (*black*)]

Urinary albumin concentration increased with severity of injury (i.e. increased histopathology grade) and with paraquat dose in rats (Fig. 4). Median urinary albumin concentrations at 24 h in 15, 30, and 60 mg/kg paraquat dose group were 35 (IQR 25–49), 45 (IQR 19–86) and 87 (IQR 26–117) μg/ml respectively.

Urinary albumin correlated strongly with uCysC (r = 0.9, *p* < 0.0001), well with uNGAL (r = 0.7, *p* < 0.0001), uKIM-1 (r = 0.6, *p* < 0.01), and uβ2M (r = 0.7, *p* < 0.001), but not with uOstP (r = 0.3, *p* < 0.05) or uClu (r = 0.3, *p* < 0.05) (Additional file 2: Figure S3). Correlations of similar magnitude were also observed after normalising the biomarker concentrations to urinary creatinine (Additional file 2: Figure S4).

#### Biomarker concentrations at 24 h in paraquat treated rats based on albuminuria

**Fig. 5:**
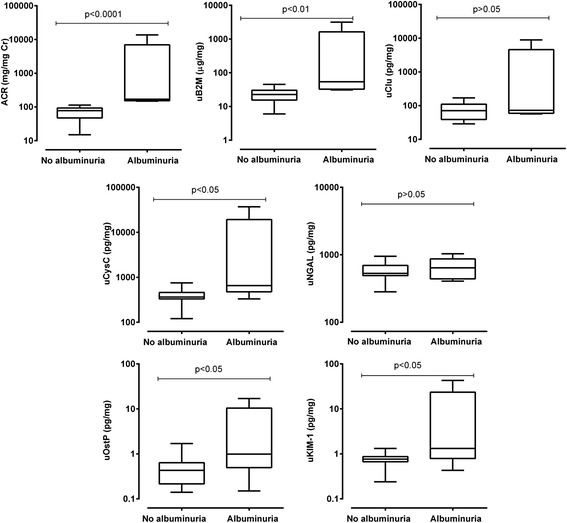
Urinary biomarker concentrations according to albuminuria in paraquat-induced nephrotoxicity in rats. This depicts urinary biomarker concentrations at 24 h stratified by albuminuria with respect to paraquat induced nephrotoxicity. Albuminuria was defined as ACR ≥115 (μg/mg Cr) which is the 95th centile values of ACR in control rats

The 95th centile ACR from control rats used to define albuminuria was 115 μg/mg Cr. Albuminuria was associated with increased concentrations of uβ2M, uCysC, uOstP, and uKIM-1 in paraquat treated rats (Fig. 5; *p* < 0.05).

## Discussion

This study demonstrated that paraquat toxicity was associated with development of albuminuria in clinical and experimental nephrotoxicity. In turn, albuminuria was associated with increased excretion of renal biomarkers and modified biomarker sensitivity in outcome prediction. The data confirm that concentrations of the low molecular weight protein biomarkers, CysC, OstP, β2M, KIM-1 and NGAL increased in the presence of albuminuria. Albuminuria was also associated with mortality. To our knowledge, this is the first study to evaluate the influence of albuminuria on excretion of all Predictive Safety Testing Consortium (PSTC) qualified urinary biomarkers.

The influence of proteinuria and albuminuria on uNGAL, uCysC and uIL-18 concentration was previously evaluated in a heterogeneous intensive care unit patient population [26] where many fold increases in uNGAL and uCysC were seen in the presence of proteinuria or albuminuria. That study and others have highlighted that proteinuria and albuminuria result in competitive inhibition of megalin-cubulin mediated reabsorption of low molecular weight urinary proteins [14, 26, 35, 36]. Thus, the increase in filtered urinary biomarker concentrations, which result from AKI, receives a contribution from impaired absorption when albumin or other proteins are also present. Nearly all low molecular weight proteins are believed to be reabsorbed by megalin and cubulin-mediated endocytosis [36].

All 50 patients included in this analysis had ingested paraquat for deliberate self-harm and hence the change in biomarker concentration was assumed to be solely due to paraquat. In contrast to the present study, that of Nejat et al. [26] recruited patients who were older and were admitted to the intensive care unit with various clinical presentations. Not surprisingly, 20% of patients in that study had a prior history of CKD, which was further limited by the use of only semi-quantitative dipstick methods to determine proteinuria [26]. The present study amplifies those observations with better quantitation of albuminuria in a younger, presumably healthier, cohort, with implications for evolving AKI biomarker research [26, 35].

An additional novel approach in this study was the recruitment of healthy young adults from the same ethnic population to define normal biomarker reference ranges. A similar approach is used to define myocardial injury based on cutoff levels of cardiac troponin obtained from healthy reference data [33, 34]. Most previous novel kidney biomarker reference ranges have been established in control patients who were sick or exposed to similar noxious insults but who did not develop AKI [18–20]. Given that biomarker levels tend to be higher in patient controls without AKI undergoing cardiac catheterization or in the ICU compared to healthy volunteers [18, 21], using patient-based control groups to report normal levels might be misleading. Such patients might have other age or disease-specific co-morbidities [18] or have transient (formerly “pre-renal”) AKI, the mild end of a continuum of renal injury [37, 38].

Our clinical study recruited healthy young adults from the same ethnic population (median age = 24, range 15–56 years) with no previous history of chronic kidney disease (CKD) or other co-morbidities. The normal biomarker range in these healthy Sri Lankan volunteers (median age = 24, range 15–56 years, Table 1 and Additional file 1: Table S1) was similar to patients who didn’t develop AKI. The sensitivity of these cutoffs for diagnosing functional AKI or predicting death was examined in patients with and without albuminuria (Fig. 3, Table 4). Excellent sensitivity (>90%) was observed for uCysC, uClu and uβ2M in diagnosing AKI and predicting death in the presence of albuminuria. However, the sensitivity for uCysC and uClu in diagnosing functional-AKI in patients who didn’t develop albuminuria was less than 1%. In the rodent studies, we demonstrated that biomarker cutoffs for predicting histopathological change in paraquat-induced nephrotoxicity increased with increasing albuminuria (Figs. 4 and 5). These differences in biomarker concentrations between albuminuric and non-albuminuric groups confirm that renal biomarker excretion is increased in the presence of albuminuria and that the cut-offs for diagnosis of AKI may differ when albuminuria is present. Since creatinine-based criteria for defining and staging AKI may not be appropriate in situations where creatinine increases independently of glomerular filtration rate as occurs early after paraquat poisoning [12], alternative definitions based on structural biomarker levels may be needed [13–17].

Recently, we [14] defined AKI utilising both functional and structural markers and calculated cutoffs for defining AKI and subsequent AKI staging. Generalising such cutoffs to define AKI is challenging due to the different non-standardised assays currently used and if other factors, for example albuminuria, influence biomarker concentrations independently of AKI [21–25]. This study demonstrated that albuminuria increases the cutoff values for outcome prediction (Table 3). If biomarkers are used to define AKI, this study suggests that over- or under-estimation of AKI incidence may occur and that quantifying urinary albumin should be considered when defining biomarker cutoffs.

Albuminuria may result from paraquat-induced glomerular damage, increasing filtration of albumin, or from tubular injury, impairing reabsorption [39–41]. As we have shown, albuminuria itself is a good diagnostic and prognostic biomarker in paraquat-induced nephrotoxicity consistent with previous studies [42–45]. However, together with other studies, this suggests that albuminuria-increased biomarker excretion may lead to an increase in the false-positive diagnosis rate for structural AKI [42, 46]. Based on these pre-clinical and clinical studies, we propose that selection of specific biomarker cutoffs for AKI diagnosis, staging and for risk prediction should factor in the presence or absence of albuminuria. Although these observations are based on a cohort with paraquat poisoning which causes both glomerular [47] and tubular injury [28], the finding is likely to be particularly relevant to young patients with pre-existing albuminuria due to other aetiologies.

### Strengths and limitations

This study has many strengths including that it is a multi-centre prospective study recruiting previously healthy young adults following a single nephrotoxic insult, with quantification of albuminuria and the establishment of biomarker ranges in healthy subjects. The study also has several limitations. The sample size of healthy control subjects was small (*n* = 63) and the 95^th^ centile values used to define structural AKI need to be validated. Nevertheless, a sensitivity analysis using alternative cut offs (97.5 or 99%) didn’t change the conclusions. Thus healthy population cutoffs to define structural AKI may have clinical utility once validated in larger populations. Further studies are clearly warranted to examine this methodology in AKI biomarker research.

## Conclusion

Albuminuria increased excretion of most low-molecular weight urinary protein biomarkers following paraquat poisoning and enhanced biomarker sensitivity in detection of functional AKI and in predicting poor outcome. Biomarker performance was reduced with altered sensitivities and increased cutoffs in the presence of albuminuria suggesting that diagnostic and predictive biomarker cutoffs need to be qualified in the presence of albuminuria.

## Additional files

Additional file 1: Table S1. Urinary functional and injury biomarkers in healthy subjects. (DOCX 12 kb)

Additional file 2: Figure S1. Biomarker profiles stratified by ACR. (DOCX 647 kb)

## Abbreviations

ACR: Albumin creatinine ratio
AKI: Acute kidney injury
AKIN: Acute Kidney Injury Network
AUC-ROC: Area under the receiver operating characteristic curves
IL-18: Interleukin-18
LMW: Low-molecular weight
NGAL: Neutrophil gelatinase-associated lipocalin
ToxAKI: Nephrotoxic AKI
uAlb: Albumin
uClu: Clusterin
uCysC: Cystatin C
uKIM-1: Kidney injury molecule-1
uOstP: Osteopontin
uTFF3: Trefoil factor-3
uβ2M: Beta-2-microglobulin
