# Supplementary material for: Nephrotoxicity-induced proteinuria increases biomarker diagnostic thresholds in acute kidney injury
Source: BMC Nephrol. 2017 Apr 3;18:122. doi: 10.1186/s12882-017-0532-7 (PMC5379711; doi:10.1186/s12882-017-0532-7)
Supplement: Supplementary file 1 — Urinary functional and injury biomarkers in healthy subjects. (DOCX 12 kb) [file 12882_2017_532_MOESM1_ESM.docx]

**Supplementary materials**

Supplementary Table 1 Urinary functional and injury biomarkers in healthy subjects (Normalised concentration to urinary creatinine)

| Urinary Biomarkers | Median and IQR | Lower reference limit (5^th^ centile) | Upper reference limit (95^th^ centile) |
| --- | --- | --- | --- |
| Total protein | 0.17 (0.12-0.28) | 0.07 | 1.6 |
| Urea | 12.3 (10.6-15) | 7 | 19 |
| Cystatin C (ng/mg) | 20.8 (12-45) | 3.2 | 71 |
| Albumin (mg/mg) | 0.004 (0.003-0.007) | 0.0002 | 0.04 |
| NGAL (ng/mg) | 13 (8.6-31.4) | 2 | 123 |
| KIM-1 (ng/mg) | 0.54 (0.38-0.77) | 0.04 | 1.2 |
| Clusterin (ng/mg) | 213 (126-286) | 45 | 423 |
| β2M (ng/mg) | 79.0 (39-112) | 9 | 166 |
| Osteopontin (ng/mg) | 1182 (492-1926) | 148 | 5408 |
| TFF3 (ng/mg) | 857 (561-1316) | 224 | 2046 |
| IL-18 (pg/mg) | 64 (30-99) | 16 | 153 |
