# Supplementary material for: Nephrotoxicity-induced proteinuria increases biomarker diagnostic thresholds in acute kidney injury
Source: BMC Nephrol. 2017 Apr 3;18:122. doi: 10.1186/s12882-017-0532-7 (PMC5379711; doi:10.1186/s12882-017-0532-7)
Supplement: Supplementary file 2 — Biomarker profiles stratified by ACR. (DOCX 647 kb) [file 12882_2017_532_MOESM2_ESM.docx]

Supplementary Figure 1 Biomarker concentrations following paraquat ingestion stratified by ACR and AKI.

*The scatter plot depicts normalised biomarker concentrations in patients according to ACR. The grey shaded area illustrate the normal range of biomarker concentrations based on data from healthy individuals (dark grey area-5th to 75th centiles; light grey area-75th to 95th centiles of the normal ranges)*

**Supplementary Figure 1**

**

**

**Supplementary Figure 2** **Correlation between absolute biomarker concentration and albumin in patients following paraquat poisoning**

**

**

Supplementary Figure 3. Correlations between absolute urinary biomarkers and urinary albumin in paraquat treated rats.





Supplementary Figure 4. Correlations between normalised urinary biomarkers and urinary albumin creatinine ratio (ACR) in paraquat treated rats.
